# Supplementary material for: Adenylsuccinate Synthetase MoADE12 Plays Important Roles in the Development and Pathogenicity of the Rice Blast Fungus
Source: J Fungi (Basel). 2022 Jul 26;8(8):780. doi: 10.3390/jof8080780 (PMC9330342; doi:10.3390/jof8080780)
Supplement: Supplementary file 1 [file jof-08-00780-s001.zip › Figure S1 and Table S1.pdf]

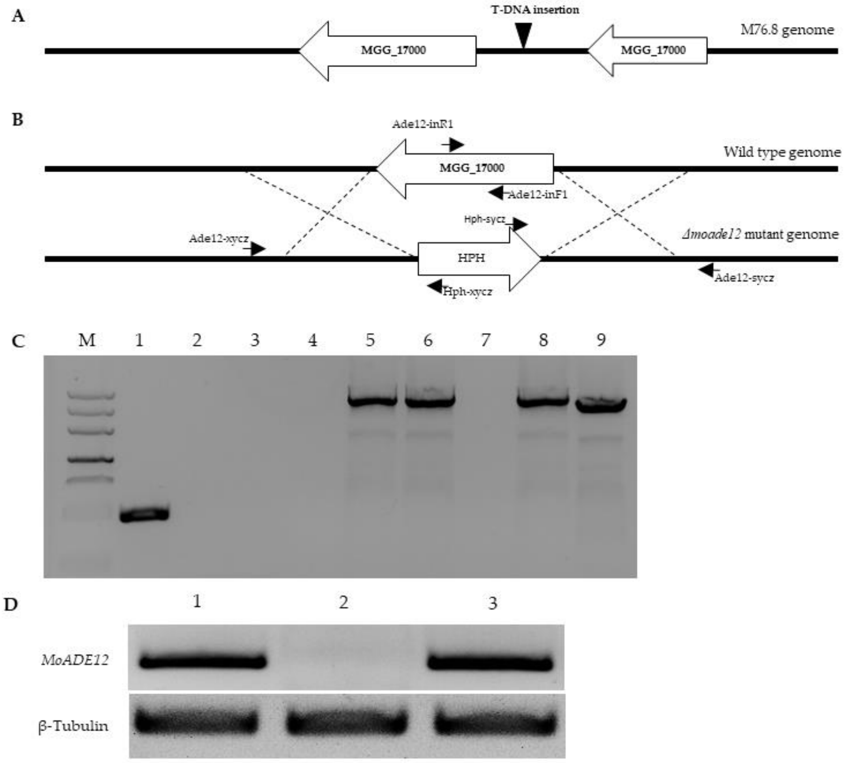

Figure S1. Generation of the *MoADE12* deletion mutants and the complement transformants. (A) Insertion site of T-DNA in the mutant M76.8 (B) Schematic diagram of *MoADE12* gene knockout. The 2.6-kb fragment including the *MoADE12* coding region was replaced with the hygromycin B (hph) cassette by homologous recombination. (C) PCR screening for gene knockout mutants. Lane M: DNA Marker III (Tiangen, Beijing, China). Lane 1-3: PCR amplification products using primer pairs Ade12-inF1/Ade12-inR1. Lane 4-6: PCR amplification products using primer pairs Ade12-sycz/Hph-sycz. Lane 7-9: PCR amplification products using primer pairs Ade12-xycz/Hph-xycz. Lane 1, 4 and 7: Guy11; Lane 2, 5 and 8: the Δ*moade12* mutant; Lane 3, 6 and 9: Another one of the *MoADE12* gene knockout mutants. (D) Expression of *MoADE12* gene in mycelia of tested strains. Lane 1: Guy11, Lane 2: the Δ*moade12* mutant, Lane 3: the reintroduction mutant *Δmoade12/MoADE12*.

Table. S1 Primers used in this study

Primers	Sequence: 5'-3'	Comments
Ade12-uF	cacacattattatggagaaactcgagtcacactgaactattcacccgaactg	pKO-MoADE12 construction
Ade12-uR	gtaccgagctcgaattcgtaatccattggtttatcttgcctgctacctg	
Ade12-dF	cctctagagtcgacctgcaggcagtgaaacttgcttttatcgcacaagacg	pKO-MoADE12 construction
Ade12-dR	gtaaaacgacggccagtgccaagcttctctccgaccaagtcactagtatagtc	
Ade12-comF	cagctatgaccatgattacgaattcacactgaactattcacccgaactg	pBAR-Ade12R construction
Ade12-comR	aacgacggccagtgccaagctcaaggtctgatacaacgcagcgtcg	
Ade12-inF1	acaacttacgtaggccttcaca	For *MoADE12* gene complementation construction
Ade12-inR1	cgcatctcggaagtctttga	
Ade12-sycz	acgcgtgcaaacacagtacc	Identification of recombination events in the Δmoade12 mutants
Hph-sycz	cgtgcaccaagcagcagatg	
Ade12-xycz	ctgcaggtctcttacccgat	Identification of recombination events in the Δmoade12 mutants
Hph-xycz	cttggttgacggcaatttcg	
Ade12q-F1	ggggatgaaggcaagggaa	*MoADE12* gene expression analysis
Ade12q-R1	gccgatcaggttcatgca	
Tub-F1	ggccaatgcggcaaccaa	MGG_00604 gene expression analysis
Tub-R1	aggacagcacggggaaca	
